# Regional and temporal variability of Indian summer monsoon rainfall in relation to El Niño southern oscillation

**DOI:** 10.1038/s41598-023-38730-5

**Published:** 2023-08-04

**Authors:** K. S. Athira, M. K. Roxy, Panini Dasgupta, J. S. Saranya, Vineet Kumar Singh, Raju Attada

**Affiliations:** 1 Centre for Climate Change Research, Indian Institute of Tropical Meteorology, Ministry of Earth Sciences, Pune, India; 2 Department of Earth and Environmental Sciences, Indian Institute of Science Education and Research Mohali, Punjab, India; 3 College of Climate Change and Environmental Sciences, Kerala Agricultural University, Thrissur, India; 4 Department of Meteorology and Oceanography, College of Science and Technology, Andhra University, Visakhapatnam, India; 5 Future Innovation Institute, Seoul National University, Siheung 15011 Seoul, Republic of Korea; 6 School of Earth and Environmental Sciences/Research Institute of Oceanography, Seoul National University, Seoul, 08826 Republic of Korea; 7 Department of Atmospheric and Space Sciences, Savitribai Phule Pune University, Pune, India; 8 Typhoon Research Center, Jeju National University, Jeju, South Korea

**Keywords:** Climate sciences, Ocean sciences

## Abstract

The Indian summer monsoon rainfall (ISMR) exhibits significant variability, affecting the food and water security of the densely populated Indian subcontinent. The two dominant spatial modes of ISMR variability are associated with the El Niño Southern Oscillation (ENSO) and the strength of the semi-permanent monsoon trough along with related variability in monsoon depressions, respectively. Although the robust teleconnection between ENSO and ISMR has been well established for several decades, the major drivers leading to the time-varying relationship between ENSO and ISMR patterns across different regions of the country are not well understood. Our analysis shows a consistent increase from a moderate to substantially strong teleconnection strength between ENSO and ISMR from 1901 to 1940. This strengthened relationship remained stable and strong between 1941 and 1980. However, in the recent period from 1981 to 2018 the teleconnection decreased consistently again to a moderate strength. We find that the ENSO–ISMR relationship exhibits distinct regional variability with time-varying relationship over the north, central, and south India. Specifically, the teleconnection displays an increasing relationship for north India, a decreasing relationship for central India and a consistent relationship for south India. Warm SST anomalies over the eastern Pacific Ocean correspond to an overall decrease in the ISMR, while warm SST anomalies over the Indian Ocean corresponds to a decrease in rainfall over the north and increase over the south of India. ﻿The central Indian region experienced the most substantial variation in the ENSO–ISMR relationship. This variation corresponds to the variability of the monsoon trough and depressions, strongly influenced by the Pacific Decadal Oscillation and North Atlantic Oscillation, which regulate the relative dominance of the two spatial modes of ISMR. By applying the PCA-Biplot technique, our study highlights the significant impacts of various climate drivers on the two dominant spatial modes of ISMR which account for the evolving nature of the ENSO–ISMR relationship.

## Introduction

Each year, the Indian subcontinent receives about 78% of its annual rainfall during the southwest monsoon season from June to September^1^. Interannual variations of the Indian summer monsoon rainfall (ISMR) are only about 9% of its mean but have significant socio-economic impact^2,3^, especially on the agriculture sector, water availability and GDP of the country^4,5^. On the interannual timescales, ISMR is affected by several ocean-atmospheric coupled climate phenomena such as the El Niño Southern Oscillation (ENSO), Indian Ocean Dipole (IOD), Pacific Decadal Oscillation (PDO), Atlantic Meridional Oscillation (AMO) and Atlantic Zonal Mode (AZM)^6–14^. ENSO being the largest tropical modulator of the Indian monsoon, is also the largest interannual climate signal in the tropics^15,16^. The changes in the zonal Walker circulation during El Niño cause anomalous subsidence over the Indian landmass, thereby suppressing the monsoon circulation and a subsequent reduction in rainfall over the Indian subcontinent^17,18^. The entire west coastal belts, monsoon zone and eastern regions are affected due to El Niño related droughts.

Generally, there are two major contributors to the interannual variability of ISMR^7^. One is the external forcing, which originates in response to climate variability and change. The second one is the internal component due to the intraseasonal activity manifested through the active and break phases of the monsoon^10,19,20^. The interannual variability of ISMR occurs partly due to external forcing and partly due to internal forcing^21^.

ISMR exhibits large spatial variability with surplus and deficit rainfall over different regions of the subcontinent. Interannual variability of ISMR is more evident when we consider the spatial variability of rainfall. Mishra et al.^22^ identified two major spatial patterns of ISMR variability and noticed that these two ISMR modes are majorly related to ENSO and the strength of the semi-permanent monsoon trough respectively. They note that the prominence of the monsoon trough is closely related to the frequency of monsoon depressions that form over the Bay of Bengal. Notably, the second pattern of ISMR has a declining strength throughout the last century (1901–2018) due to the weakening of monsoon circulation and the declining number of monsoon depressions^23–27^. As a result, there is a reduction in rainfall over the core monsoon areas of central-eastern India and the west coast of India^23,24^. However, their study does not explore how these variability and associated teleconnections manifest regionally, over different parts of the country for different time periods. The strength of the monsoon trough and frequency of depression is also related to the post-ENSO sea surface temperature (SST) conditions. Chowdary et al.^28^ found that El Niño events result in the warming of the north Indian Ocean during the subsequent summer. This warming is primarily driven by air-sea interactions occurring within the tropical Indian Ocean. While these studies are helpful in gaining overall insights into the ENSO–ISMR relationship, the regional variability and its long-term changes are less understood^29–32^. Since the effect of ENSO is not the same for different regions of the country, understanding the regional ENSO–ISMR relationship is important for identifying and improving monsoon forecast skills also.

The inverse relationship between ENSO–ISMR is the primary source of predictability of ISMR^32–34^. However, the ENSO–ISMR inverse relationship shows a weakening trend in the recent decade after the 1980s owing to several factors such as increased surface warming over Eurasia^35^, strengthening and poleward shift of the jet streams over the North Atlantic^36^, increased greenhouse gas concentration^37^ and shift in the surface wind circulation pattern over the Indo-Pacific region^38^. Importantly, the ENSO–ISMR inverse relation also varied spatially over the past century. Mahendra et al. (2021)^39^ studied this spatial variation and suggested that the ENSO–ISMR relationship is modified in different epochs with a steady relationship in northern central India and southern peninsula and a decreasing relationship in central and east India. This spatial variability is linked to anomalous upward motion associated with Indian Ocean Dipole (IOD) and the westward extension of low-level cyclonic circulation from the Western-North Pacific region.

In this study, our primary focus is to examine the changes in the relationship between ENSO and ISMR, during the summer monsoon months (JJAS). We specifically investigate how the major drivers of ISMR influence this relationship. Our first objective is to analyze the associations between the dominant spatial modes of ISMR and various climate processes, including IOD, PDO, North Atlantic Oscillation (NAO), Quasi–Biennial Oscillation (QBO), Interdecadal Pacific Oscillation (IPO), AMO, and Atlantic Niño. We aim to understand how these different climate modes locally impact the ENSO–ISMR relationship. Our second objective aims to provide a comprehensive overview of the reasons behind the changing ENSO–ISMR relationship across different regions of the country (south, central, and north India).

## Results and discussion

### Dominant modes of ISMR variability and its drivers

**Figure 1:**
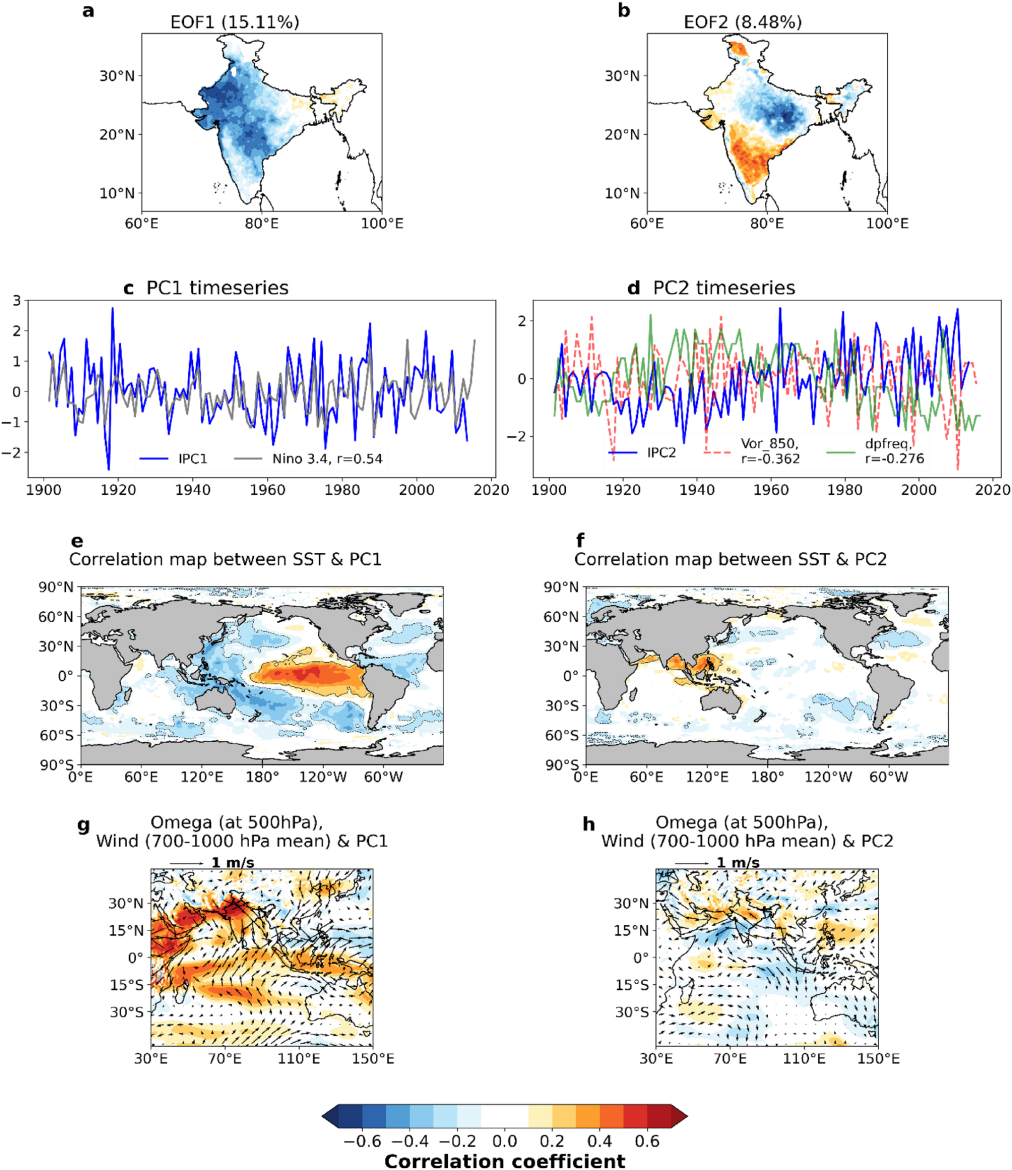
(**a**) EOF1 (**b**) EOF2 of standardised ISMR. (**c**) time series of EOF1 represented as PC1 correlated with Niño 3.4 time series (**d**) time series of EOF2 represented as PC2 correlated with the time series of depression frequency and time series of vorticity at 850 hPa. (**e**) and (**f**) Correlated global SST pattern of ISMR EOF1 and EOF2 respectively (**g**) and (**h**) Correlation of PC1 and PC2 with both wind (700-1000 hPa mean) and vertical velocity at 850 hPa respectively. Colour scale denotes correlation coefficient and standardized anomaly of rainfall. This figure is created using Python 3.8.0 software (https://www.python.org/downloads/release/python-380/).

The dominant modes of spatial variability pattern of ISMR are isolated by employing EOF analysis on standardised rainfall data^22^. Figure 1a–b shows the first two leading EOFs of standardised ISMR from 1901 to 2018. The first mode shows rainfall anomalies all over India, particularly the central and north-western parts of India and explains 15% of the total variance. A dipole pattern is seen in the second mode of EOF in which positive rainfall anomalies are present in Gangetic plains and negative anomalies are seen in south India or vice versa (Fig. 1b). A reduction in rainfall is also observed over the core monsoon areas of central-eastern India and most parts of west coast as observed by Roxy et al.^23^ and Vishnu et al.^24^. Darshana et al.^40^ noticed positive rainfall anomalies over the western-southern peninsular India and negative rainfall anomalies over the eastern Indo-Gangetic plains and attributed it to the Indo-western Pacific Ocean Capacitor (IPOC) mode. EOF2 explains 8.5% of the total variance. The time series corresponding to these first two modes (principal components) are named as PC1 and PC2 (Fig. 1c, d). PC1 indicates the first mode of EOF. The Niño 3.4 time series is also plotted along with PC1 and there is a strong correlation between them with a coefficient value of 0.54 which is statistically significant at the 95% confidence level (Fig. 1c). PC2 indicates the second mode of EOF and shows an increasing trend (Fig. 1d). This means that the negative and positive rainfall anomaly pattern over Gangetic plains and southern peninsula is increasing respectively. Along with this PC2 time series, the vorticity at 850 hPa averaged over 80°E–100°E, 10°N–30°N (representing the strength of the monsoon trough) and the frequency of monsoon depression are represented in Fig. 1d. The correlation of PC2 with vorticity at 850 hPa and depression frequency is found out to be -0.36 and -0.28 respectively which is statistically significant at the 95% confidence level.

In Fig. 1e, PC1 shows a positive correlation with SST over Niño 3.4 region, implying that a reduction in rainfall over India in EOF1 mode is associated with the warm SSTs over the Niño 3.4 region signifying the influence of El Niño in causing a decrease in monsoon rainfall. In Fig. 1f, PC2 shows a positive correlation with the SSTs over north Indian Ocean (Arabian Sea and the Bay of Bengal) and the South China Sea. This indicates that a reduction in rainfall over the Gangetic plains and an increase in rainfall over the southern peninsula is related to warm SSTs over the north Indian Ocean (Arabian Sea, Bay of Bengal) and South China Sea. These warm SSTs further indicate the influence of Indian Ocean warming in bringing about this condition on long term trends in ISMR.

In order to identify the major drivers of ISMR variability corresponding to EOF1 and EOF2, correlation analysis is carried out for PC1 and PC2 with mean winds at 700 – 1000 hPa to study the associated low-level circulations and omega at 500 hPa. In Fig. 1g, PC1 (indicating reduced ISMR) is positively correlated with the omega at 500 hPa which means that the decrease in rainfall over India is related to the increased omega at 500 hPa. It can also be observed from the same figure that PC1, when correlated with the low-level winds, exhibits a weak circulation pattern related to the reduced rainfall. Similarly, in Fig. 1h, PC2 is negatively correlated with the omega at 500 hPa over the southern peninsula, implying that an increase in rainfall there is related to a decrease in omega. In addition, PC2 is positively correlated with the omega at 500 hPa over the Gangetic plains signifying that a decrease in rainfall over the Gangetic plains is related to the increase in omega. Also, PC2 when correlated with low level winds show anomalous westerlies, that can carry more moisture from the Arabian Sea to the Bay of Bengal favouring the formation of monsoon depressions.

### The effect of different climate modes on regional ISMR variability

**Figure 2:**
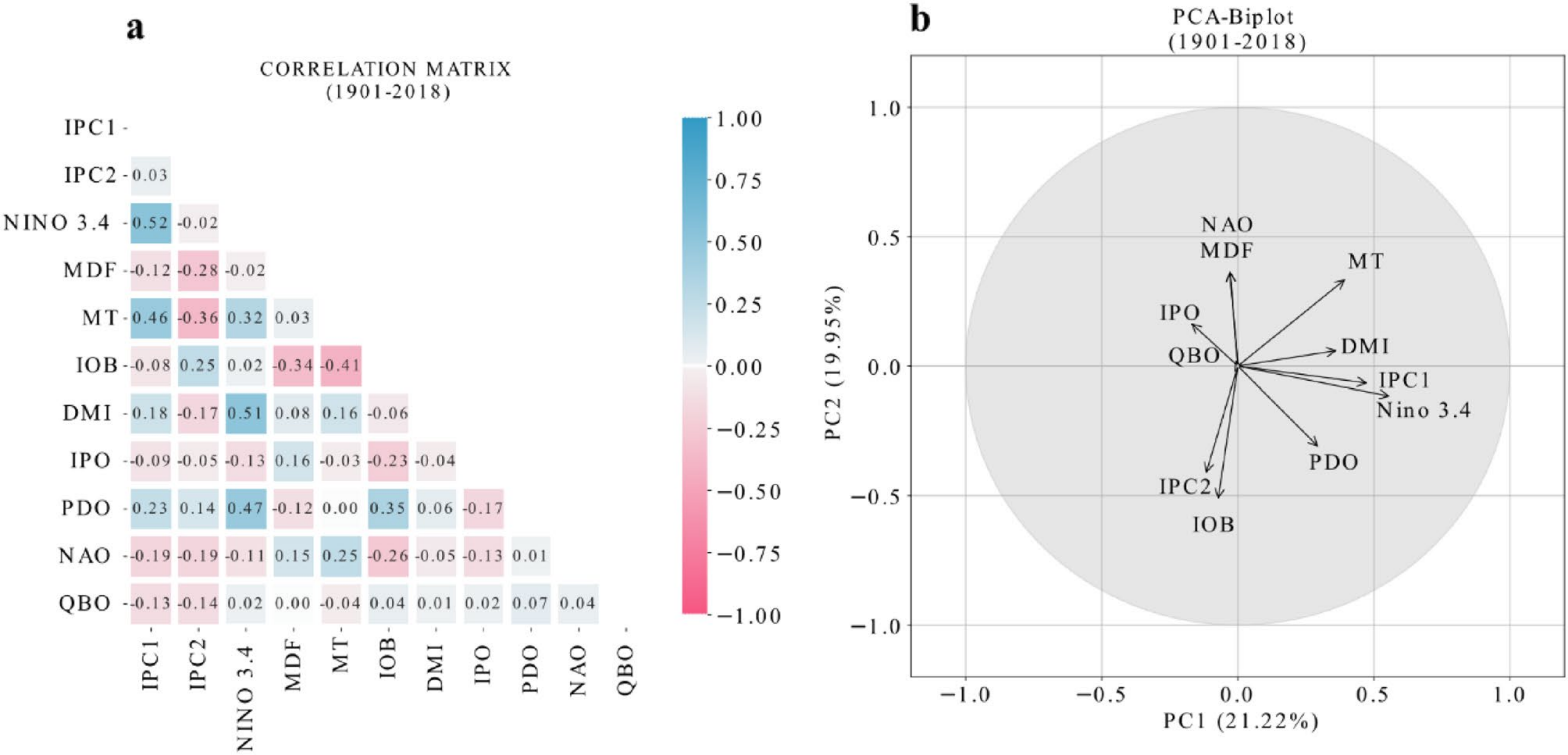
(**a**) Correlation matrix and (**b**) PCA-Biplot of different climate mode indices i.e., (IPC1) (PC1 with ISMR), (IPC2) (PC2 with ISMR), Niño 3.4 index, yearly monsoon depression frequency (MDF), monsoon trough (MT) index defined as vorticity anomalies at 850 hPa averaged over 80˚E-100˚E, 10˚N-30˚N, Indian Ocean Basin (IOB) mode index calculated from the first EOF of Indian Ocean SST, Dipole Mode Index (DMI) for Indian Ocean Dipole, tri-pole index for Interdecadal Pacific Oscillation (IPO), Pacific Decadal Oscillation (PDO) index, index for North Atlantic Oscillation (NAO), Quasi Biennial Oscillation (QBO) index for the period 1901 –2018. The axes represent the principal components from the PCA of the correlation matrix. PC1 and PC2 explain 21.22% and 19.95% variance of the total variance respectively (together 41% variability). This figure is created using Python 3.8.0 (https://www.python.org/downloads/release/python-380/).

Apart from ENSO, various other climate modes ranging from interannual to multidecadal timescales, such as IOD^7,41,42^, IOB^43^, PDO^44,45^, Interdecadal Pacific Oscillation (IPO)^46^, QBO^47^, NAO^48,49^, Atlantic Niño^50^, and AMO^51,52^, also influence ISMR. To explore the role of these different climate drivers on the ISMR variability, correlation matrix and biplot analysis are used. The major drivers, ENSO and the strength of the monsoon trough have 0.5 (Fig. 1c) and − 0.36 (Fig. 1d) correlation coefficient values with the ISMR. While these two factors are dominant in regulating ISMR variability, this is also an indication that there are other factors that lead to ISMR variation. Hence the various climate modes are taken into consideration and their role in the ISMR variability is analysed using the correlation matrix and biplot. We find that the correlation values between the first two ISMR variability patterns and AMO and Atlantic Niño index are weak and insignificant (*p* > 0.05). Therefore, these two climate modes are not discussed further in our study. Regardless, a subsequent mode of ISMR variability linked to the Atlantic Niño related variability cannot be entirely dismissed. Figure 2a represents the correlation matrix of different climate indices. This gives the correlation between different climate indices with PC1 and PC2. The PC1 and PC2 in the PCA biplot is different from the PC1 and PC2 of ISMR variability. Here in the biplot PC1 and PC2 refer to a leading mode of covariability between different climate modes. Since PC1 and PC2 are two independent processes their correlation is very low (-0.03). The PCA-biplot (Fig. 2b), represents the interrelationship between different climate modes. In two dimensional biplot space, an arrow represents a variable and its length denotes the percentage of variance. The unit circle implies the maximum correlation value one. There are two independent processes ISMR PC1 and ISMR PC2. The processes that cluster around ISMR PC1 are related to each other and those which cluster around ISMR PC2 are interrelated. The processes close to x-axis are grouped under one category and the ones close to y-axis are grouped under other. We observe that PC1 is connected with ENSO, IOD, PDO and IPO. Meanwhile, PC2 is associated with the NAO, IPO, PDO and IOB mode index along with monsoon trough (MT) strength and depression frequency (MDF). MT is connected to both PC1 and PC2. Physically it means that MT is related to both ENSO and internal factors related to monsoon variability. PDO, IPO, and MT contribute to both PC1 and PC2 spatial patterns of rainfall. The biplot space does not explain the QBO well (the length of arrow is short). From the biplot we can infer that the MDF vector and the PDO vector has an out of phase relationship. This finding aligns with the study by Vishnu et al.^13^ that over the past seven decades the monsoon depressions that form over the Bay of Bengal has an out of phase relationship with PDO due to the variation in the relative humidity. The natural climate variability is driven by various climatic oscillations and understanding the physical mechanism for the variation in the spatial and temporal scale variability of ISMR is still complex as it is influenced by large scale atmospheric, oceanic and coupled climate phenomena^53^.

**Figure 3:**
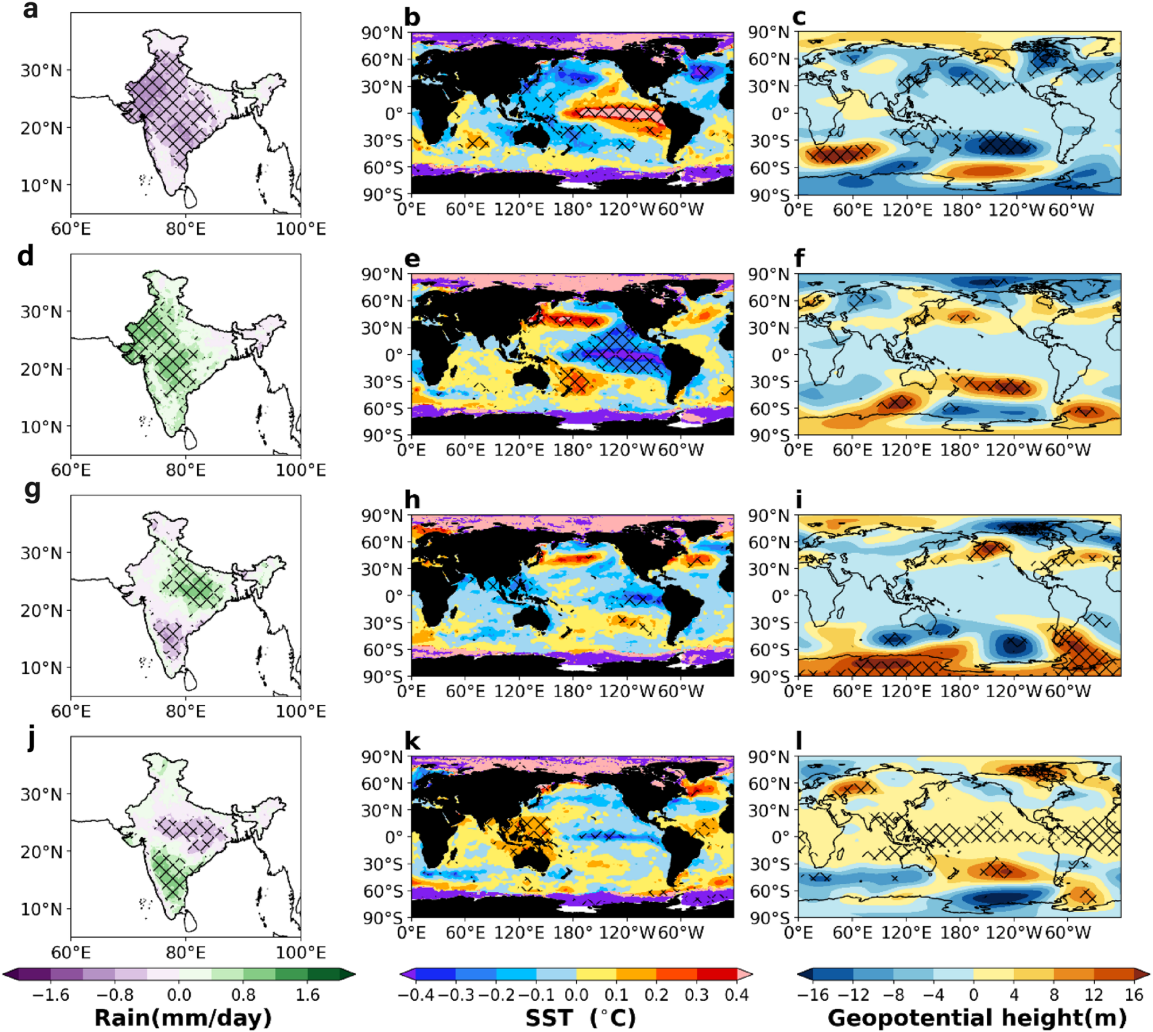
Composites of seasonal (JJAS mean) ISMR, SST and Z500 (geopotential height at 500 hPa) anomalies for the years when (**a–c**) PC1 greater than its positive standard deviation value, (**d–f**) PC1 less than its negative standard deviation value, (**g–i**) PC2 greater than its positive standard deviation, (**j–l**) PC2 less than its negative standard deviation. This figure is created using Python 3.8.0 software (https://www.python.org/downloads/release/python-380/).

To provide the physical interpretation of the biplot, we have done a composite analysis considering the phases of the EOF modes (PC1 and PC2). Here Fig. 3a–c represents the composites of ISMR, SST and geopotential height when PC1 is greater than its positive one standard deviation values and vice-versa when PC1 is in the negative phase (Fig. 3d–f). We notice that the ENSO and PDO patterns are present in the ISMR PC1 composites. Similarly, Fig. 3g–i represent the composites when PC2 is greater than its positive standard deviation values and vice-versa when PC2 is in the negative phase (Fig. 3j–l). Here a north–south dipole pattern in the rainfall anomaly is observed, indicative of strength of monsoon trough and variability in monsoon depressions. In the composites of ISMR PC2, the role of NAO and IOB is prominent.

### Changing ENSO-ISMR relationship and regional rainfall variability

**Figure 4:**
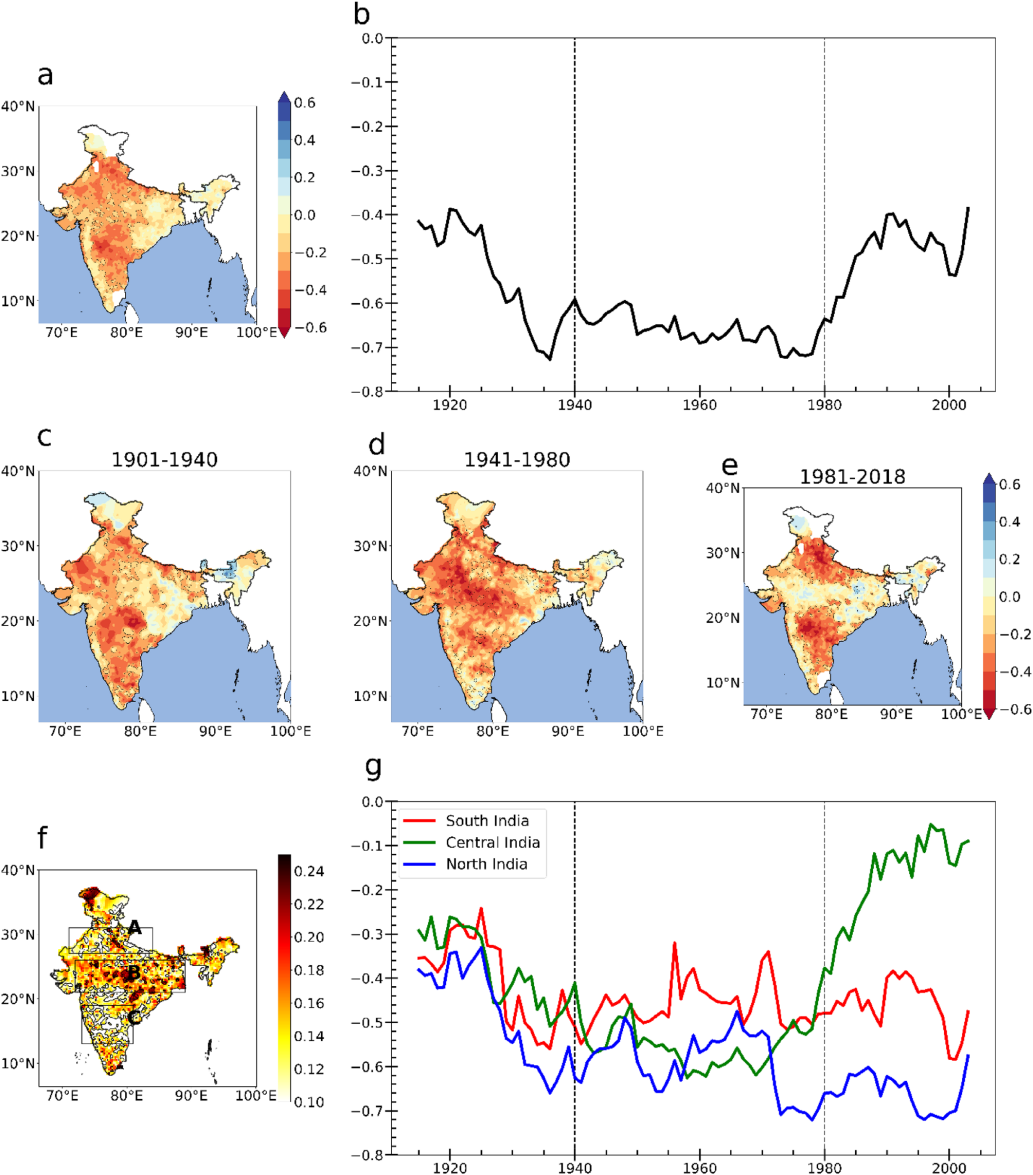
(**a**) spatial map of correlation between ISMR and ENSO from 1901 to 2018 (**b**) 30 year running correlation between ISMR and Niño 3.4 SST. (**c–e**) spatial map of correlation between ISMR and ENSO during 1901–1940, 1941–1980 and 1981–2018. (**f**) The variability (standard deviation) in the running correlation between ISMR and Niño 3.4 time series (**g**) 30-year running correlation between the mean rainfall at the three boxes (north, central and south) and Niño 3.4 SST. This figure is created using Python 3.8.0 software (https://www.python.org/downloads/release/python-380/).

In order to understand the association between ENSO and ISMR, a correlation analysis is carried out between their representative indices. Figure 4a shows the spatial correlation between Niño 3.4 SST (JJAS (June, July, August, September)) anomaly and ISMR anomaly, during the period 1901 – 2018. Overall, there is a negative correlation for most parts of India while a very weak negative correlation exists for the Western Ghats, east India and northeast India. In south India, central India and parts of north India the correlation of ENSO and rainfall varies between -0.2 to -0.6 which is statistically significant at the 95% confidence level.

To see if this ENSO–ISMR relationship is consistent or varying, a running correlation is done which helps us to understand the evolution of ENSO–ISMR relationship with time. Figure 4b represents the 30-year running correlation of ENSO–ISMR relationship for the period 1901 to 2018. The correlation between the ENSO and ISMR varies between -0.4 to -0.7. Based on the change in the ENSO–ISMR relationship, the entire period from 1901 to 2018 is divided into three periods; 1901–1940, 1941–1980 and 1981–2018. From 1901 the correlation between ENSO and ISMR started rising from -0.41 until 1940. The ENSO–ISMR relationship then became stable for the period 1941 to 1980 with a very strong negative correlation between the values -0.6 to -0.7. Then after 1980, the correlation began to decline from a coefficient value of -0.6 to -0.4 which indicates that after 1980, the ENSO–ISMR relationship is weakening, which is in agreement with Seetha et al.^32^. Theses epochs also coincides with positive, negative and positive PDO phases respectively.

The Fig. 4c–e shows the spatial map of the correlation between ISMR anomalies and Niño 3.4 region SST anomalies (JJAS) for different periods. This shows that the ENSO–ISMR relationship not only shows temporal variability but also has a strong spatial variability. From 1901 to 1940, the relationship was prominent over south India, parts of the west and north India. In the middle period from 1941–1980 the relationship between the ENSO and ISMR was strong over most parts of the country as seen from high negative correlation values. After 1980, the relationship between ENSO and ISMR weakens except for south and north India. Central India and Western Ghats are devoid of a negative interrelationship and a weak positive correlation can also be observed. Chakravorty et al.^54^ pointed out that in the recent years during the development phase of El Niño, central India displays weak El Niño ISMR relationship while for south India a strong negative correlation is observed. Comparing the ENSO–ISMR relationship for all the three periods, it can be said that for south India the relationship is stable for all the three periods. For central India the ENSO–ISMR relationship has been decreasing in the recent period and for north India there has been a gradual increase in the relationship from 1901 to 2018.

This variability in the ENSO–ISMR relationship for different regions of India can be visualised in the spatial map of running correlation. In Fig. 4f, along with the standard deviation, a 30-year running correlation is carried out between JJAS monthly rainfall anomaly at each grid point in the Indian region and Niño 3.4 SST anomaly during JJAS from 1901–2018 to distinctly point out the regions of variability. The standard deviation of the running correlation denotes the variability in correlation over time. We identify the regions where the variability in the correlation is large and small and accordingly three boxes are selected (Fig. 4f). These three boxes, corresponding to the north (71˚E–84˚E, 27˚N–31˚N), central (72˚E–89 ˚E, 21˚N–26˚N) and south India (73˚E–81˚E, 13˚N–19˚N) represent moderate, largely variable and low correlation respectively with the Niño 3.4 time series. We observe low variability in the ENSO–ISMR relationship in the southern peninsula. A moderate variability is observed in the north Indian region. The highest variability in the ENSO–ISMR relationship is observed in the central Indian region.

To explore the ENSO–ISMR relationship for three different regions during different periods of time, a time series analysis of the running correlation for the three regions is carried out. Figure 4g shows the 30-year running correlation between the mean rainfall over the north, central and south regions and Niño 3.4 SST. Over south India, the ENSO–ISMR relationship is consistent over the last century (1901–2018) as shown by near-constant running correlation over this region (the correlation value varies between -0.3 to -0.6). The relationship between ENSO and the north India rainfall has increased with time as seen from the correlation values increasing from -0.4 to -0.7 during the same period. The ENSO–ISMR relationship exhibits the highest variability over central India. From 1901 to 1940 the correlation between rainfall of central India and Niño 3.4 was weak (approximately -0.3 to -0.5). During the period 1941 to 1980, a strong correlation was observed (approximately -0.5 to -0.6). Again, during the recent period (1981 to present), the correlation with Niño 3.4 SST has become weak (approximately between -0.1 to -0.3). Before 1980, the correlation varied similarly at all three regions with increasing values till 1980 suggesting the strong ENSO–ISMR relationship. But after 1980, there is a significant change in the ENSO–ISMR relationship for north, central and south India where there is an increasing, weakening and stable correlation respectively. For north India, the relationship between ENSO and ISMR becomes strong after 1980 as seen by an increase in the correlation values. For central India, the ENSO–ISMR relationship has been weakening in recent years. South India shows a stable negative correlation ranging between -0.3 to -0.6 for the entire period, indicating that the ENSO–ISMR relationship remains almost constant in this region throughout the study period.

**Figure 5:**
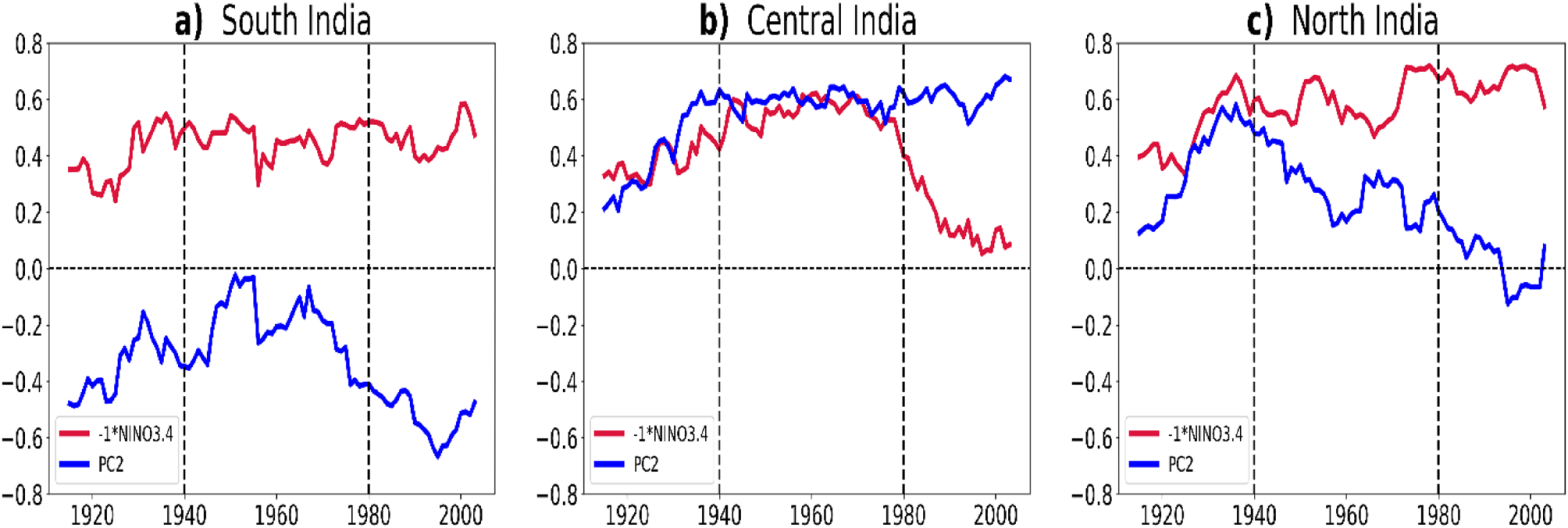
30 year running correlation of (**a**) south India box rainfall (**b**) central India box rainfall (**c**) north India box rainfall with Niño 3.4 SST (red line) and Monsoon trough/depressions related variability represented by PC2 (blue line). This figure is created using Python 3.8.0 software (https://www.python.org/downloads/release/python-380/).

The Fig. 5 shows the 30-year running correlation for rainfall over each of the three regions over India, with respect to the two drivers, ENSO and the monsoon trough. -1*Niño 3.4 represents La Niña condition and hence shows a positive correlation with rainfall of south India, central India and north India. For south India (Fig. 5a) the influence of both the factors are dominant which means that south India rainfall is dependent on ENSO variability as well as the strength of monsoon trough and related variability in monsoon depressions. In Fig. 5b, for central India, the contribution by ENSO and monsoon trough and depressions are increased up to 1980, and then the contribution by ENSO started declining drastically. However, the influence of monsoon trough and depressions remained strong. Thus, a decline in the number of monsoon depressions^24^ can significantly affect the central India rainfall. (The decreasing influence of ENSO and increasing influence of monsoon trough strength and depression related variability for central India can be easily interpreted by comparing both the time series of running correlation. Hence Niño 3.4 is multiplied by -1). In sharp contrast to central India, the influence of ENSO on the rainfall in north India is increasing (Fig. 5c) and has become stronger in the recent period, while the impact of monsoon trough and depressions on the rainfall is decreasing in this region. This may be due to the decreasing reach of the monsoon depression into the north Indian region in recent decades^24,55^. Mahendra et al.^39^ also found similar spatial inhomogeneity in ENSO–ISMR relationship for north, central and south India but has linked this spatial variability as a response to IOD and westward extension of low-level circulation from Western North Pacific. They observed stable and stronger ENSO**-**ISMR relationship in all the epochs for both north and south India. But our study shows an increasing relationship for north India and a stable relationship for south India and a decreasing relationship for central India. We attribute this variation in ENSO–ISMR relation for different parts of the country to monsoon trough and related variability in monsoon depressions. Since the PC2 is also significantly related to the phases of NAO other than monsoon trough and depression, we also investigate the changing NAO–ISMR relationship (Supplementary Fig S1). This plot shows that like ENSO–ISMR relationship, NAO–ISMR relation is maximum variable (changed) over central India, specifically over central-east India. Similar to the Fig. 4g, Supplementary Fig. S1(e) represent the time evolution of NAO-ISMR relation over north, south and central India. NAO–ISMR relation was found to be relatively strong during 1901–1940, 1981–2018 and had opposite sign. The relation was weak during 1941–1980 when ENSO dominance was strong. NAO–ISMR correlation for three period 1901–1940, 1941–1980 and 1981–2018 indicates that during 1901–1940 and 1981–2018 a north–south and east–west dipole structures are present respectively. However, NAO–ISMR relation was weak during negative PDO phase during 1941–1980. Variability in NAO–ISMR relation is maximum over central-India region where variability in ENSO–ISMR relation is also maximum. NAO–ISMR relation is strong in positive PDO phases and weak in negative PDO phases as opposite to ENSO–ISMR relationship. Importantly, Goswami et al.^56^ also emphasised the significance of NAO on the predictability of ISMR due to the complexity of relationship between north Atlantic SST and ISMR and the role of NAO on ISMR can be linked to the influence of extratropical Rossby waves on ISMR.

**Figure 6:**
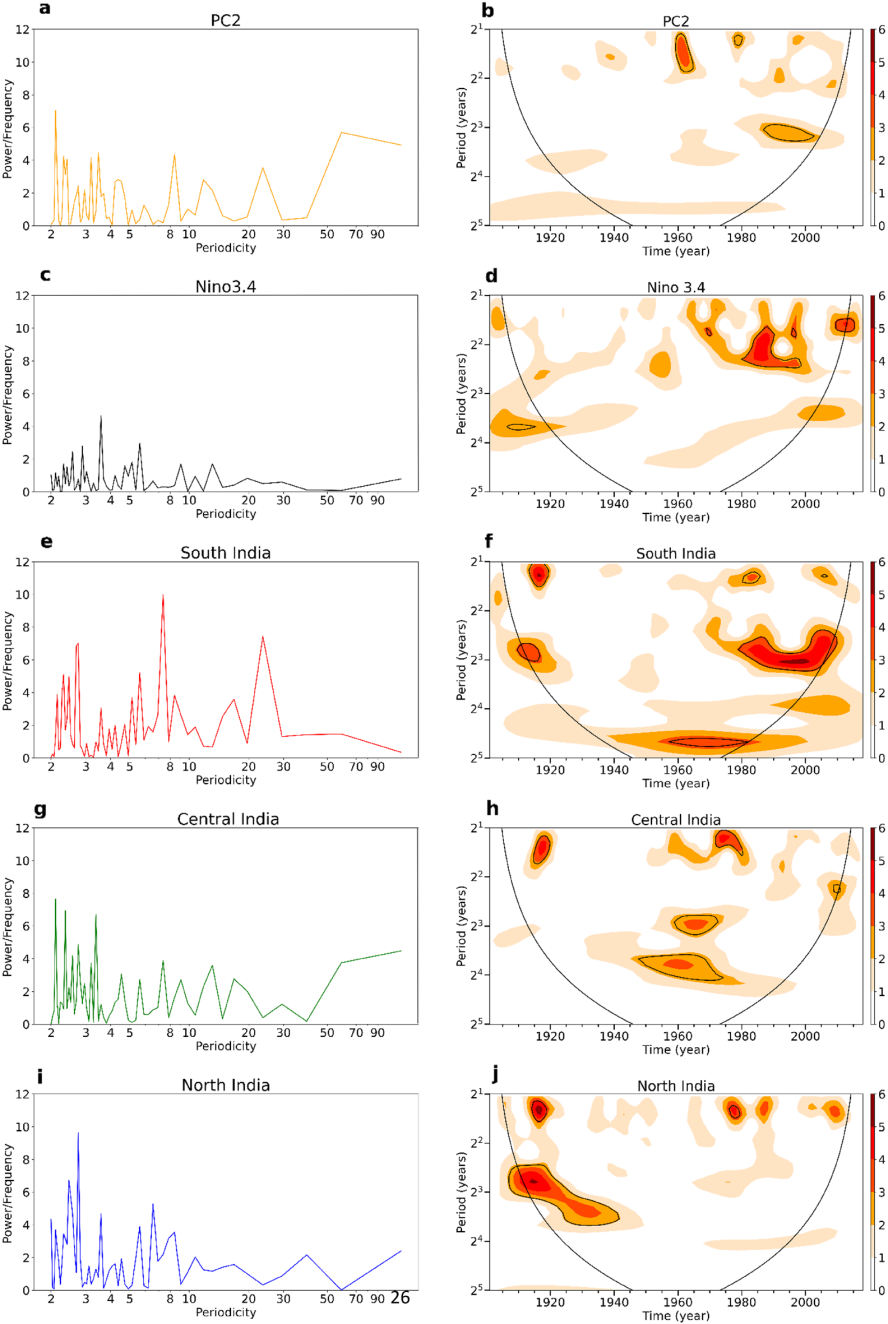
Power spectrum and wavelet of: ISMR PC2 (**a**) showing 2–4 years and a decadal variability, (**b**) 2–4 years of variability present in 1960s and decadal variability after 1980s, Niño 3.4 index (**c**) showing 2–7 years and a decadal variability (**d**) 2–7 years of variability seen in 1980s, JJAS rainfall in South India box (**e**) showing 2–7 years and a decadal variability (**f**) 2–7 years of variability seen in 1980s, Central India box (**g**) showing 2–4 years and a decadal variability (**h**) 2–4 years of variability present in 1960s and North India box (**i**) showing only 2–7 years of variability (**j**) 2–7 years of variability seen in 1910–1940s. This figure is created using Python 3.8.0 software (https://www.python.org/downloads/release/python-380/).

The influence of the two major drivers on the rainfall over the three regions are also evident in the power spectrum and wavelet analysis. The periodicity present in the time series and the corresponding timescale can be identified from the power spectrum and wavelet analysis respectively (Fig. 6). For PC2, the power spectrum shows both 2–4 years periodicity and a decadal variability (Fig. 6a). From the wavelet analysis it can be seen that those 2–4 years of variability are seen in the year 1960 and the decadal variability is seen after 1980 (Fig. 6b). The power spectrum of Niño 3.4 SST (Fig. 6c) indicates a 2–7 years periodicity and the decadal variability associated with ENSO. The wavelet analysis of Niño 3.4 SST (Fig. 6d) shows that 2–7 years periodicity is more prominent after 1960. The decadal variability is more prominent for PC2 compared to that of Niño 3.4.

In order to identify the frequency associated with the time series of north, central and south India regions, the power spectrum is drawn (Fig. 6a, c, e, g, i). Wavelet analysis is carried out to identify the time scale in which these frequencies are present (Fig. 6b, d, f, h, j). For south India, a periodicity of 2 to 7 years (Fig. 6e) is prominent during the years up to 1920 and then from 1980 to 2000 (Fig. 6f) which is similar to that of Niño 3.4 SST showing the relationship with ENSO. A 2–7 year variability is observed from the power spectrum of both Niño 3.4 and south India rainfall which is prominent after the 1960’s (from the wavelet analysis of Niño 3.4 and south India rainfall). This implies that south India rainfall is related to ENSO. A decadal variability is also seen for south India indicating a connection with the decadal variability represented in PC2. Central India shows a frequency signal between 2 to 4 years (Fig. 6g) from 1910 to 1920 and 1960 to 1980 (Fig. 6h). The power spectrum of central India resembles that of PC2 which means that the rainfall over central India is dependent on PC2. For north India 8 years of periodicity (Fig. 6i) can be seen during the years 1910 to 1940 (Fig. 6j). Comparing it with the power spectrum and wavelet analysis of Niño 3.4 SST and PC2 it is seen that north India rainfall is dependent on Niño 3.4 SST and not on PC2 because it does not exhibit decadal variability. It is clear from this analysis that the rainfall of south India shows a relation with both ENSO and monsoon trough and depressions, central India rainfall exhibits relation with only monsoon trough and depressions and north India rainfall has its relation with ENSO only. Hence power spectrum and wavelet analysis confirm the influence of the two major drivers on north, central and south India shown in running correlation.

## Conclusion

The current study re-examines the dominant spatial variability patterns of ISMR and their evolution with time over different parts of the country, and investigates the drivers of this variability. Here, we investigate how the changing influences of ISMR drivers affect its variability, with a primary focus on the changing influence of ENSO on ISMR. There are two major spatial patterns of ISMR which explain about 24% of the total interannual variability. The first mode (PC1) implies reduced rainfall across the Indian subcontinent, especially the central and western parts of India, accounting for 15.11% of the total variability. The second mode (PC2) indicates a dipole pattern with reduced rainfall over the Indo-Gangetic plain and increased rainfall over south India accounting for 8.48% of the total variability. The PC1 pattern of ISMR is significantly influenced by ENSO, IOD, PDO, IPO, and variation in the monsoon trough. While the PC2 pattern is significantly associated with NAO, IOB, PDO, IPO, MDF and the strength of the monsoon trough.

It is noteworthy that there are two distinct groups of climate processes, namely ENSO and IOD influencing PC1-like spatial rainfall patterns, and NAO and IOB favouring PC2-like spatial rainfall patterns. As previously discussed in other studies, the role of NAO on ISMR can be linked to the influence of extratropical Rossby waves on ISMR (Goswami 2022). Interestingly, PDO, IPO, and variation in monsoon trough strength (MT) contribute to both PC1 and PC2 spatial patterns of rainfall. The decadal variation of monsoon depression frequency and its association with PDO have been explored by Vishnu et al.^13,24^. Similarly, both leading modes of ISMR variability can be affected by the strength of the monsoon trough.

Furthermore, we examine the changes in the ENSO–ISMR relationship over the past century and the role of two dominant spatial variability patterns in driving these changes. As previously discussed by Mahendra et al.^39^, we also observe significant spatio-temporal variability in the ENSO–ISMR relationship. The relationship between ENSO and ISMR has not remained consistent throughout the period from 1901 to 2018. We notice that the ENSO–ISMR inverse relationship started getting stronger from 1901 to 1940, became stable from 1941 to 1980 and then the relationship has weakened in the recent epoch (1981 onwards). Theses epochs also coincides with positive, negative and positive PDO phases respectively. However, this change in ENSO–ISMR relationship is spatially non-uniform. We find that over south India there is no considerable variation in the ENSO–ISMR relationship. Whereas over north India the ENSO–ISMR relationship is becoming strong in recent decades and has shown significant multidecadal variation. On the contrary, association between the central India rainfall and ENSO has diminished in the recent decades. We find that the role of monsoon trough and depression related variability has emerged as the primary cause of rainfall variability over central India surpassing the role of ENSO related variability. Earlier, we demonstrated that NAO and IOB are significantly associated with monsoon trough and depression-related variability. Based on this, we also find an increasing relationship between NAO and ISMR over the central Indian region. For the rainfall over south India, the influence of ENSO and strength of monsoon trough and variability related to monsoon depressions have been consistent over the entire period. Over north India, rainfall variability is increasingly dependent on ENSO, while the role of the monsoon trough and depression is decreasing. This may be due to the decreasing reach of the monsoon depression into the north Indian region in recent decades^24,55^. Our study highlights the changing influence of monsoon drivers over different parts of the country. However, the current study does not discuss the specific mechanisms involved in the changing ISMR variability influenced by climate patterns such as NAO, but recommends it for future research.

## Materials and methods

In the present study, we use correlation analysis to examine the relationship between ENSO and ISMR, EOF analysis to identify the major drivers of ISMR variability, power spectrum analysis to distinguish the frequency present in the time series for the rainfall of different regions and wavelet analysis to identify the corresponding timescale in which these frequencies are present in the time series. Standardised rainfall data at each grid point is used for the EOF analysis at each grid point. The statistical significance of the analysis carried out here is ascertained following the student t-test. A Principal Component Analysis (PCA) biplot analysis is carried out to identify the influence of different phenomena on the two major variability patterns of ISMR. The concepts of PCA biplot technique were explained by Jollife and Cadima^57^. The PCA biplot represents both observations and variables in a two-dimensional bivariate plane and denotes the direction of maximum variability. In a PCA biplot, perpendicular vectors represent independent processes. The amplitude of the vector represents the variance of the process. The processes which are close to each other are statistically similar. The PCs and all analysis are based on JJAS season.

### Materials used

Rainfall data is obtained from the India Meteorological Department (IMD). Monthly and daily gridded rainfall data with a resolution of 0.25˚ × 0.25˚^58^ is used for the analysis from 1901–2018 during the June to September months, covering 118 years. The SST data used is the Hadley Centre Sea Ice and Sea Surface Temperature data set (HadISST) obtained from the Met Office Hadley Centre observations datasets with a resolution of 1˚ × 1˚^59^ for the same period. NOAA-CIRES-DOE Twentieth Century Reanalysis (V3) is used for retrieving the monthly horizontal and vertical velocity, meridional wind component, zonal wind component and geopotential height dataset. This dataset has 1˚ × 1˚ resolution. The Niño 3.4 index, Dipole Mode Index (DMI) for IOD, Tripole Index (TPI) which tracks the decadal variability of SST associated with IPO and the indices for PDO, NAO and Quasi-Biennial Oscillation (QBO) are obtained from the World Meteorological Organisation (WMO) and National Oceanic and Atmospheric Administration (NOAA) climate indices archive. Depression frequency data is obtained from the IMD data archive. We derived the Indian Ocean Basin (IOB) mode index from the first Empirical Orthogonal Function (EOF) of SSTs in the Indian Ocean (40˚E–80˚E, 20˚S–40˚N) and it represents the first mode of interannual variability in the tropical Indian Ocean^60^. IOB mode is characterised by basin wide warming or cooling and can influence the climate of the surrounding regions^61^. IOB index peaks during the post-ENSO years. The Niño 3.4 index is defined as the area averaged SST anomalies in the Niño 3.4 region (5˚S–5˚N, 170˚W–120˚W). DMI measures the strength of the IOD represented by the anomalous SST difference between the western equatorial Indian Ocean (50˚E–70˚E and 10˚S–10˚N) and the south-eastern equatorial Indian Ocean (90˚E–110˚E and 10˚S–0˚N). The TPI index is derived from the SST anomaly differences between the central equatorial Pacific (170˚E–90˚W and 10˚S–10˚N) and the northwest (140˚E–145˚W and 25˚N–45˚N) and southwest Pacific (140˚E–145˚W and 25˚N–45˚N). PDO index is defined as the leading principal component of North Pacific monthly sea surface temperature variability poleward of 20˚N. NAO index consists of a north–south dipole in atmospheric pressure anomalies, with one centre located over Greenland and the other centre of opposite sign spanning the central latitudes of the North Atlantic between 35˚N and 40˚N. The index is based on a rotated principal component analysis of monthly standardized 500mb height anomalies in the North Atlantic poleward of 20˚N. The QBO index from 1948 onwards is based on the zonal averaged (5˚N – 5˚S) u wind at 30 hPa pressure level. Before 1948, the QBO index is taken from the Free University of Berlin (FUB) database. All these indices are averaged for JJAS season. The definition of various indices is summarised in Table 1.

**Table 1:** The indices of different climate modes and their definitions.

Index	Definition
IOB	First EOF of SSTs in the Indian Ocean
Niño 3.4	Area averaged SST anomalies in the Niño 3.4 region (5˚S–5˚N, 170˚W–120˚W)
DMI	Anomalous SST difference between the western equatorial Indian Ocean (50˚E–70˚E and 10˚S–10˚N) and the south-eastern equatorial Indian Ocean (90˚E–110˚E and10˚S–0˚N)
TPI	SST anomaly differences between central equatorial Pacific (170˚E–90˚W, 10˚S–10˚N) and the northwest (140˚E–145˚W, 25˚N–45˚N) and southwest pacific (140˚E–145˚W, 25˚N–45˚N)
PDO	Leading principal component of North Pacific monthly SST variability poleward of 20˚N
NAO	North–south dipole in atmospheric pressure anomalies, with one centre located over Greenland and the other centre of opposite sign spanning the central latitudes of the North Atlantic between 35˚N and 40˚N
QBO	Based on zonal averaged (5˚N–5˚S) u wind at 30 hPa pressure level

### Supplementary Information

Supplementary Information.

## Data Availability

We used daily and monthly gridded rainfall data to study the rainfall variability^58^. Hadley Centre Sea Ice and Sea Surface Temperature (HadISST) data set is obtained from the Met Office Hadley Centre^59^ and is available at https://www.metoffice.gov.uk/hadobs/hadisst/data/download.html. Monthly vertical velocity, meridional wind component, zonal wind component and geopotential height data is obtained from NOAA-CIRES-DOE Twentieth Century Reanalysis (V3) available at https://psl.noaa.gov/data/gridded/data.20thC_ReanV3.html. Various climate indices such as the Niño 3.4 index, Dipole Mode Index (DMI), Tripole Index (TPI), the indices for Pacific Decadal Oscillation (PDO), North Atlantic Oscillation (NAO) and Quasi – Biennial Oscillation (QBO) are obtained from the World Meteorological Organisation (WMO) and National Oceanic and Atmospheric Administration (NOAA) climate indices archive available at https://psl.noaa.gov/data/climateindices/list/. Depression frequency data is available at cyclone e-atlas on IMD site http://14.139.191.203/.

## Code availability

The codes used in the current analysis are available at https://github.com/paninidasgupta/ENSO-Monsoon-Relation/tree/main.
