# Author Correction: Regional and temporal variability of Indian summer monsoon rainfall in relation to El Niño southern oscillation

**DOI:** 10.1038/s41598-023-42501-7

**Published:** 2023-09-15

**Authors:** K. S. Athira, M. K. Roxy, Panini Dasgupta, J. S. Saranya, Vineet Kumar Singh, Raju Attada

**Affiliations:** 1 Centre for Climate Change Research, Indian Institute of Tropical Meteorology, Ministry of Earth Sciences, Pune, India; 2 Department of Earth and Environmental Sciences, Indian Institute of Science Education and Research Mohali, Punjab, India; 3 College of Climate Change and Environmental Sciences, Kerala Agricultural University, Thrissur, India; 4 Department of Meteorology and Oceanography, College of Science and Technology, Andhra University, Visakhapatnam, India; 5 Future Innovation Institute, Seoul National University, Siheung, Seoul, 15011 Republic of Korea; 6 School of Earth and Environmental Sciences/Research Institute of Oceanography, Seoul National University, Seoul, 08826 Republic of Korea; 7 Department of Atmospheric and Space Sciences, Savitribai Phule Pune University, Pune, India; 8 Typhoon Research Center, Jeju National University, Jeju, South Korea

## Introduction

Correction to: *Scientific Reports* https://doi.org/10.1038/s41598-023-38730-5, published online 04 August 2023

In the original version of this Article, Panini Dasgupta and J. S. Saranya were incorrectly affiliated with ‘Seoul National University, Seoul, South Korea’. The correct affiliations are listed below:

**Panini Dasgupta:**

- Centre for Climate Change Research, Indian Institute of Tropical Meteorology, Ministry of Earth Sciences, Pune, India.
- Department of Meteorology and Oceanography, College of Science and Technology, Andhra University, Visakhapatnam, India.
- ‘Future Innovation Institute, Seoul National University, Siheung 15011, Seoul, Republic of Korea’

**J. S. Saranya:**

- Centre for Climate Change Research, Indian Institute of Tropical Meteorology, Ministry of Earth Sciences, Pune, India.
- College of Climate Change and Environmental Sciences, Kerala Agricultural University, Thrissur, India
- ‘School of Earth and Environmental Sciences/Research Institute of Oceanography, Seoul National University, Seoul 08826, Republic of Korea’

The original Article and the accompanying Supplementary Information file have been corrected.

### Supplementary Information

Supplementary Information.

