# Supplementary material for: Author Correction: Regional and temporal variability of Indian summer monsoon rainfall in relation to El Niño southern oscillation
Source: Sci Rep. 2023 Sep 15;13:15299. doi: 10.1038/s41598-023-42501-7 (PMC10504304; doi:10.1038/s41598-023-42501-7)
Supplement: Supplementary file 1 — Supplementary Information. [file 41598_2023_42501_MOESM1_ESM.docx]

**Regional and Temporal Variability of Indian Summer Monsoon Rainfall in relation to El Niño Southern Oscillation**

**Athira K S^1,2,3^, M K Roxy^1^, Panini Dasgupta^1,4^, Saranya J S^1,2^, Vineet Kumar Singh^1,5^, Raju Attada^3^**

^1^Centre for Climate Change Research, Indian Institute of Tropical Meteorology, Pune, India

^2^College of Climate Change and Environmental Sciences, Kerala Agricultural University, Thrissur, India

^3^Indian Institute of Science Education and Research Mohali, Punjab, India

^4^Department of Meteorology and Oceanography, College of Science and Technology, Andhra University, Visakhapatnam, India

^5^Department of Atmospheric and Space Sciences, Savitribai Phule Pune University, Pune, India

**Corresponding author**: Athira K S

Email: [athirakskp@gmail.com](mailto:athirakskp@gmail.com)

**Supplementary Information**


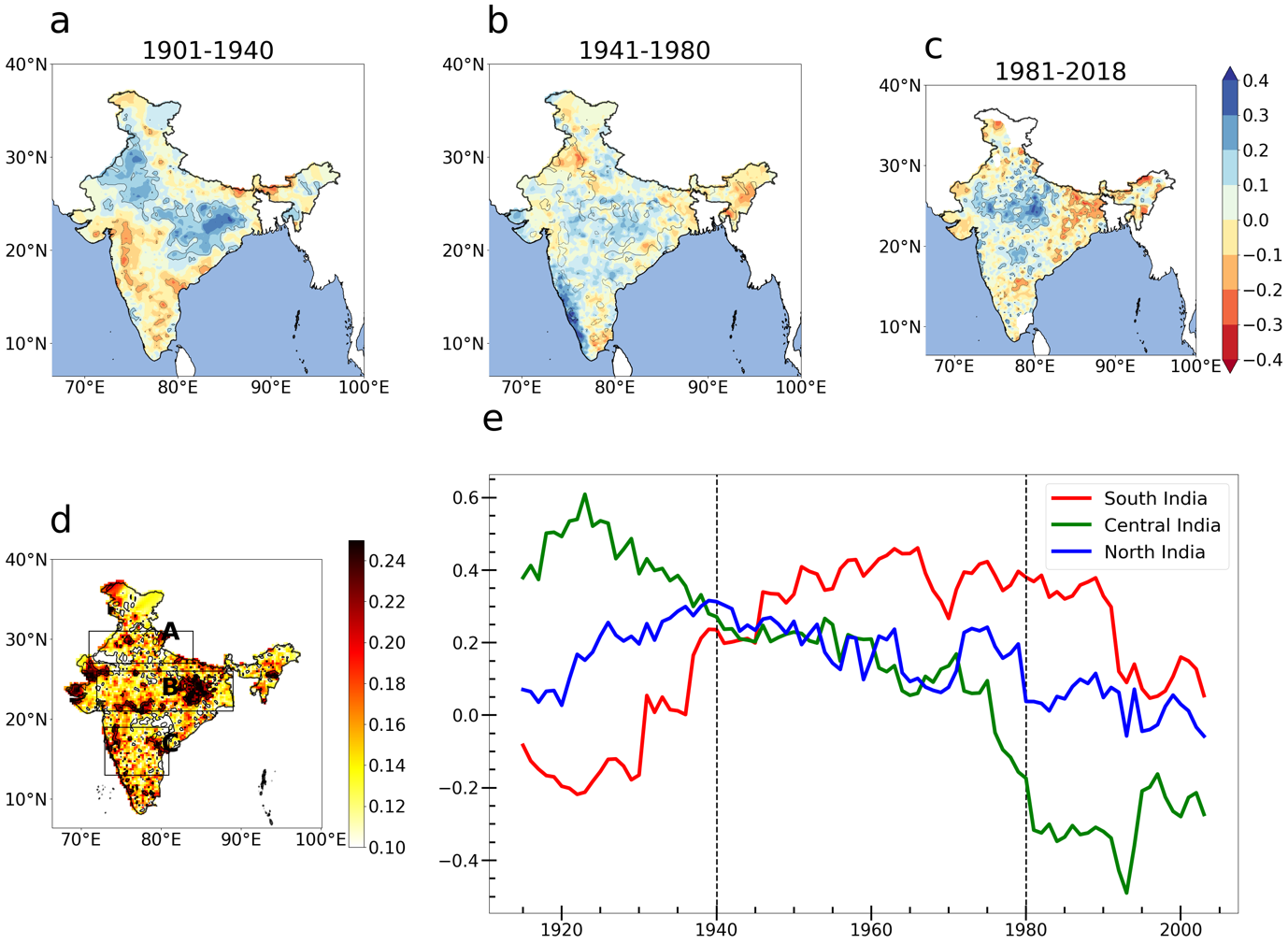


**Figure. S1** (**a, b and c)** spatial map of correlation between ISMR and NAO during 1901 – 1940, 1941 – 1980 and 1981 – 2018. **(d)** The variability (standard deviation) in the running correlation between ISMR and NAO time series **(e)** 30-year running correlation between the mean rainfall of the three boxes (north, central and south) and NAO index. This figure is created using Python 3.8.0 software (<https://www.python.org/downloads/release/python-380/).>
